# In this month’s *Bulletin*

**DOI:** 10.2471/BLT.16.000216

**Published:** 2016-02-01

In the editorial section, John C Reeder & Winnie Mpanju-Shumbusho (78) argue for a sustained research effort on poverty-related diseases. Emma Allanson et al. (79) announce changes in the way perinatal mortality is categorized by the *International statistical classification of diseases and related health problems.*

In the news section, Gary Humphreys (82‒83) reports on moves to assign adult ratings to films that show actors smoking. W Aubrey Webson (84‒85) tells Fiona Fleck about gains made for people with disabilities.

## Ghana

## Counting maternal deaths

Joseph Adomako et al. (86–91) assess the feasibility of community-based maternal mortality surveillance in rural areas.

## Turkey

## Smoking in public buildings

Ana Navas-Acien et al. (92–102) evaluate compliance with anti-smoking legislation.

## Brazil

## Dying from neglected diseases

Francisco Rogerlândio Martins-Melo et al. (103–110) estimate mortality from neglected tropical diseases.

## Global

## Water and sanitation

Jeanne Luh & Jamie Bartram (111–121) correlate access to improved water and sanitation with socioeconomic indicators in 73 countries.

## Preventing iodine deficiencies

Trach Duc Tran et al. (122–129) measure changes in households’ access to iodized salt.

## Improving care for small babies

Grace J Chan et al. (130–141) review the factors that help and hinder kangaroo care.

## Balancing two jobs

Barbara McPake et al. (142–146) consider dual public and private sector practice by health workers.

## Building clinical trial capacity

Thelma Tupasi et al. (147–152) describe a global capacity-building programme to develop a new treatment for multidrug-resistant tuberculosis.

## Polio vaccines and mobile phones

Abdul Momin Kazi & Lubna Ashraf Jafri (153–154) discuss the use of mobile phones in polio eradication.

## Better maternal mortality data

Rob E Dorrington & Debbie Bradshaw (155–156) call for more investment in civil registration and vital statistics.

